# Supplementary figures and images for: System identification of signaling dependent gene expression with different time-scale data
Source: PLoS Comput Biol. 2017 Dec 27;13(12):e1005913. doi: 10.1371/journal.pcbi.1005913 (PMC5760096; doi:10.1371/journal.pcbi.1005913)

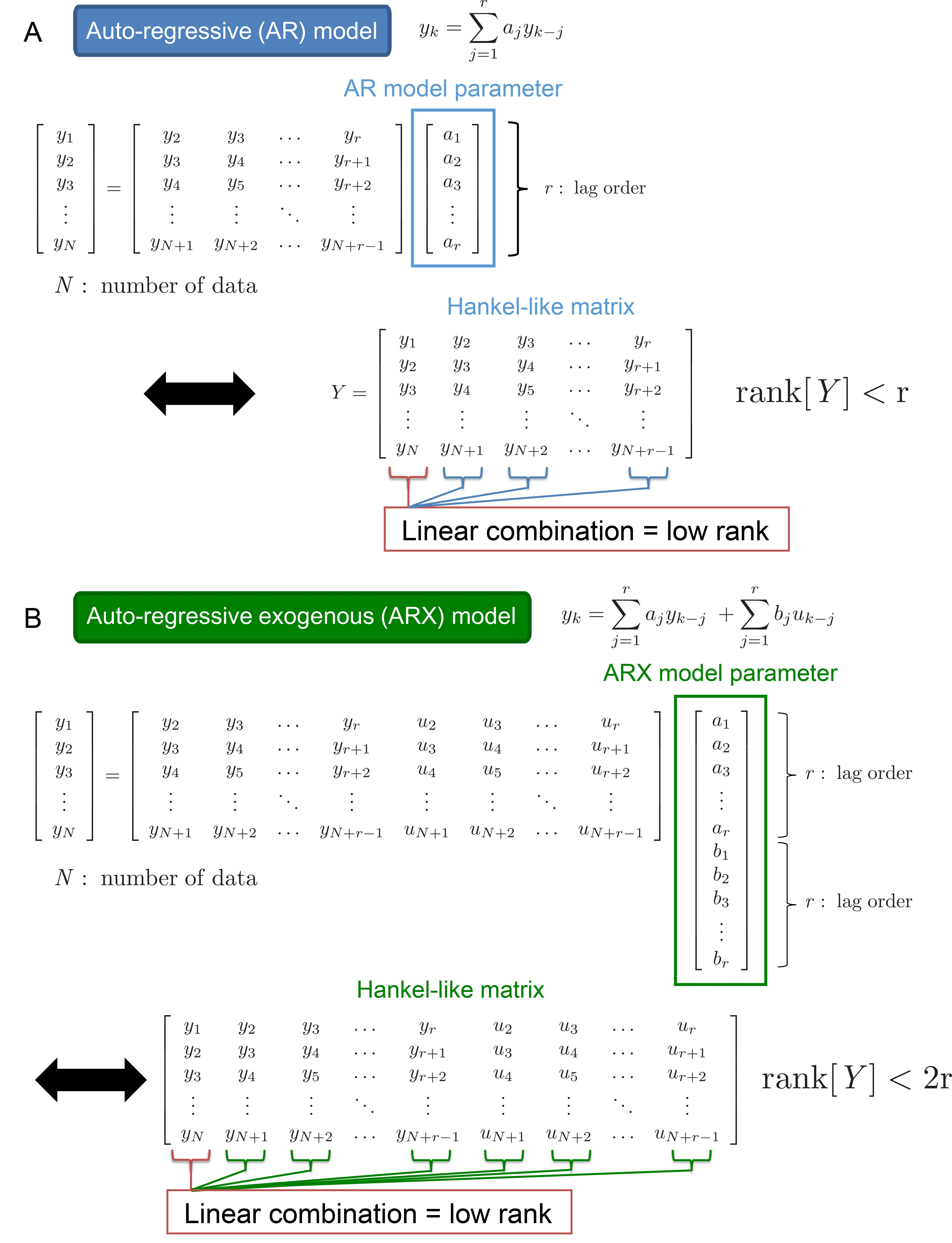

Supplement: S1 Fig — (A) Estimation of AR parameters by rank minimization of Hankel-like matrix. When the signal y follows an AR model, y1 is represented by the linear sum from y2 to yr where r is lag order. This relationship can be expressed using AR parameters as the first row of the above matrix equation. Similarly, y2 is represented by the linear sum from y3 to yr+1 as expressed in the second row. Therefore, for number of data N, the matrix equation is established as shown in the above formula. Given that this matrix equation holds, in the Hankel-like matrix below, the first column is represented by the linear sum of the second and subsequent columns, indicating that the matrix is a low-rank matrix. Note that Hankel-like matrix is a one in which the same components are entered from the lower left to the upper right here. Therefore, assuming that y follows an AR model, in case that there is missing data in y, it can be recovered by minimizing the rank of this Hankel-like matrix. Once the Hankel-like matrix is obtained, AR parameters can be estimated by solving the above matrix equation. (B) Estimation of ARX parameters by rank minimization of a Hankel-like matrix. In the case of ARX model with input u added, the similar Hankel-like matrix composed of y and a Hankel-like matrix composed of u, and the matrix equation of the above equation are established. Assuming that y and u follow the ARX model, missing data in y and u can be recovered by minimizing the rank of this Hankel-like matrix, and ARX parameters can be estimated by solving the above matrix equation. Note that this method can be applicable for multiple inputs by increasing the number of Hankel-like matrices composed of u. (TIF) [file pcbi.1005913.s001.tif]

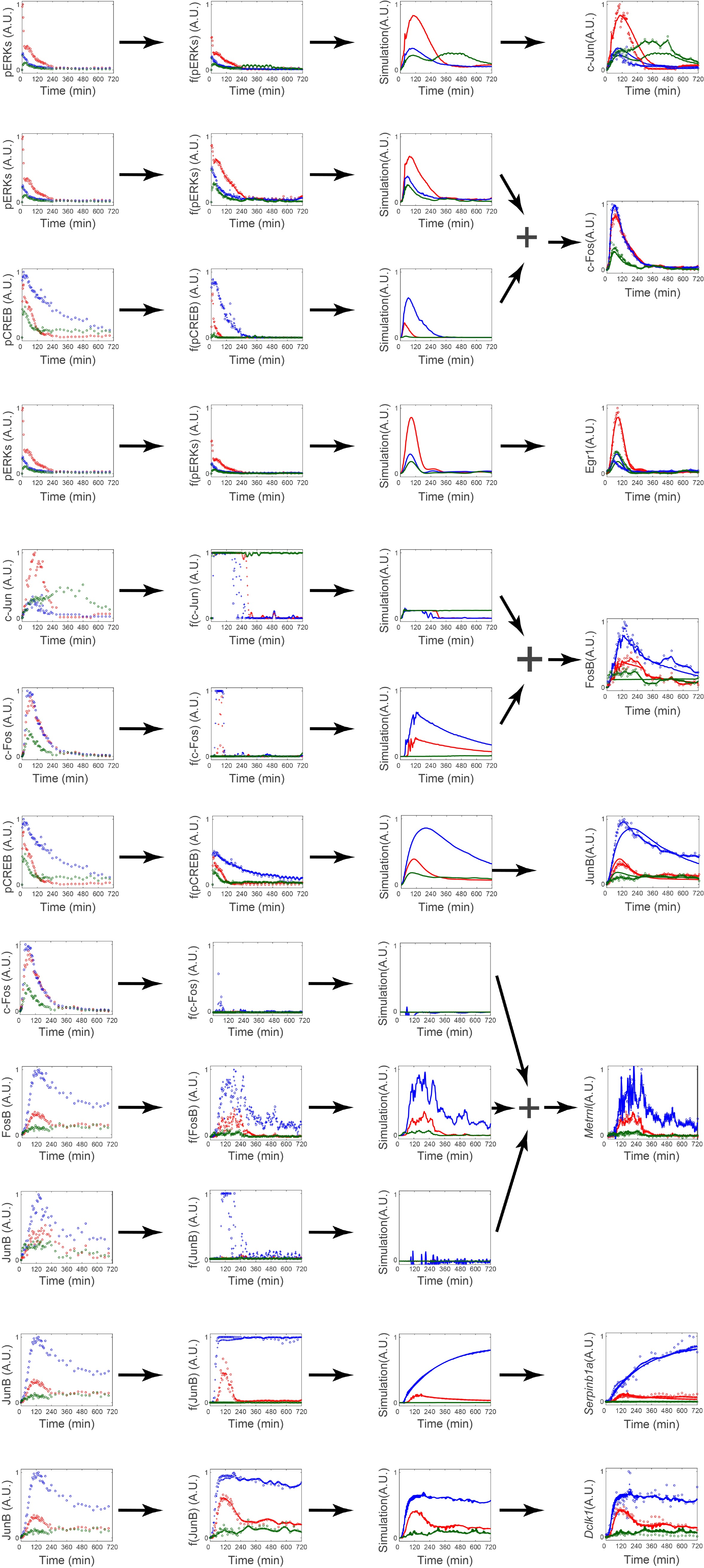

Supplement: S2 Fig — (TIF) [file pcbi.1005913.s002.tif]

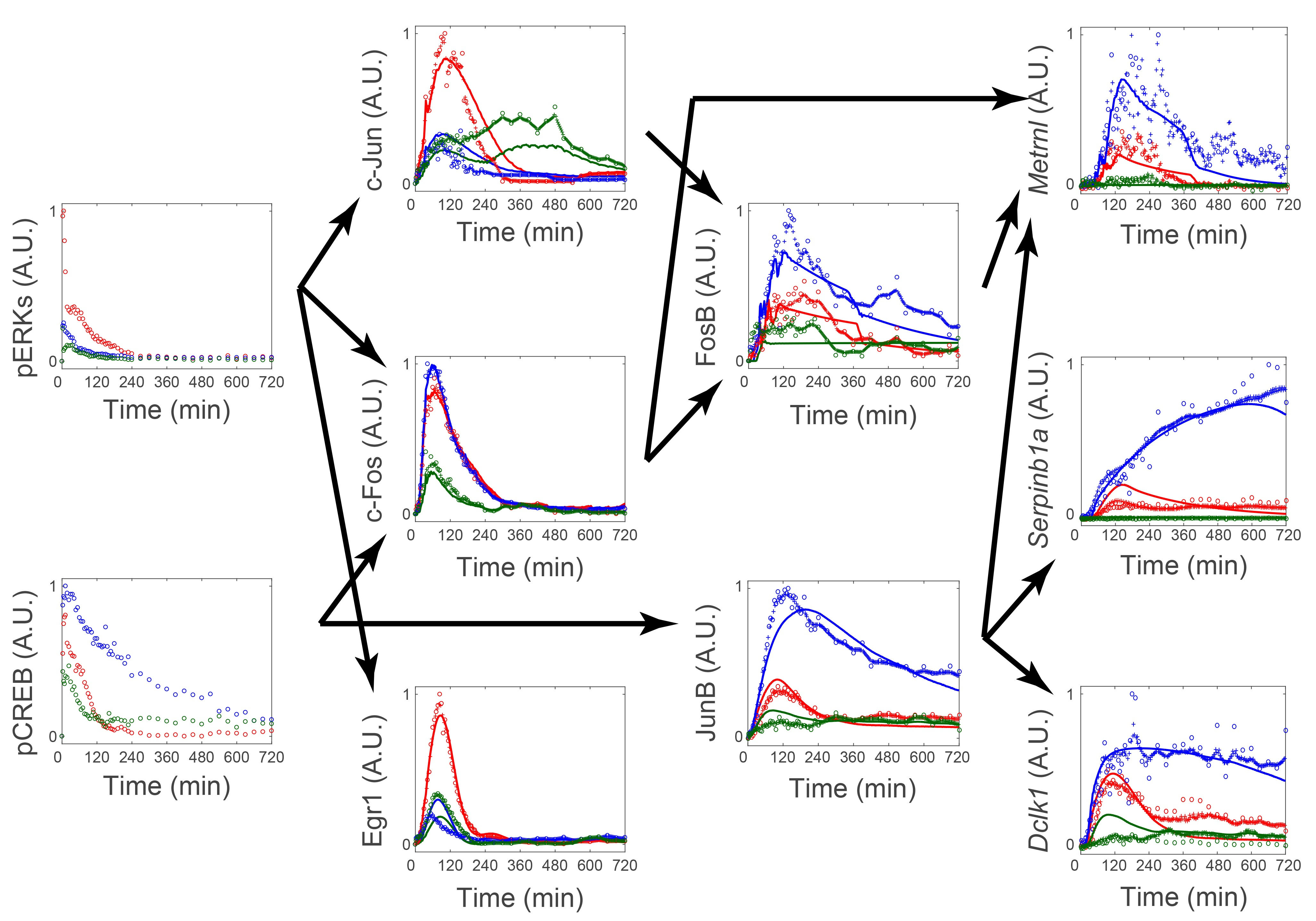

Supplement: S3 Fig — Solid lines, the simulation results by the integrated NARX model identified in Fig 5 using only pERK and pCREB as Inputs (see “Simulation of the integrated NARX model” section in Materials and methods); dots, experimental data; pluses, the recovered signal data; red, NGF stimulation; blue, PACAP stimulation; green, PMA stimulation. (TIF) [file pcbi.1005913.s003.tif]

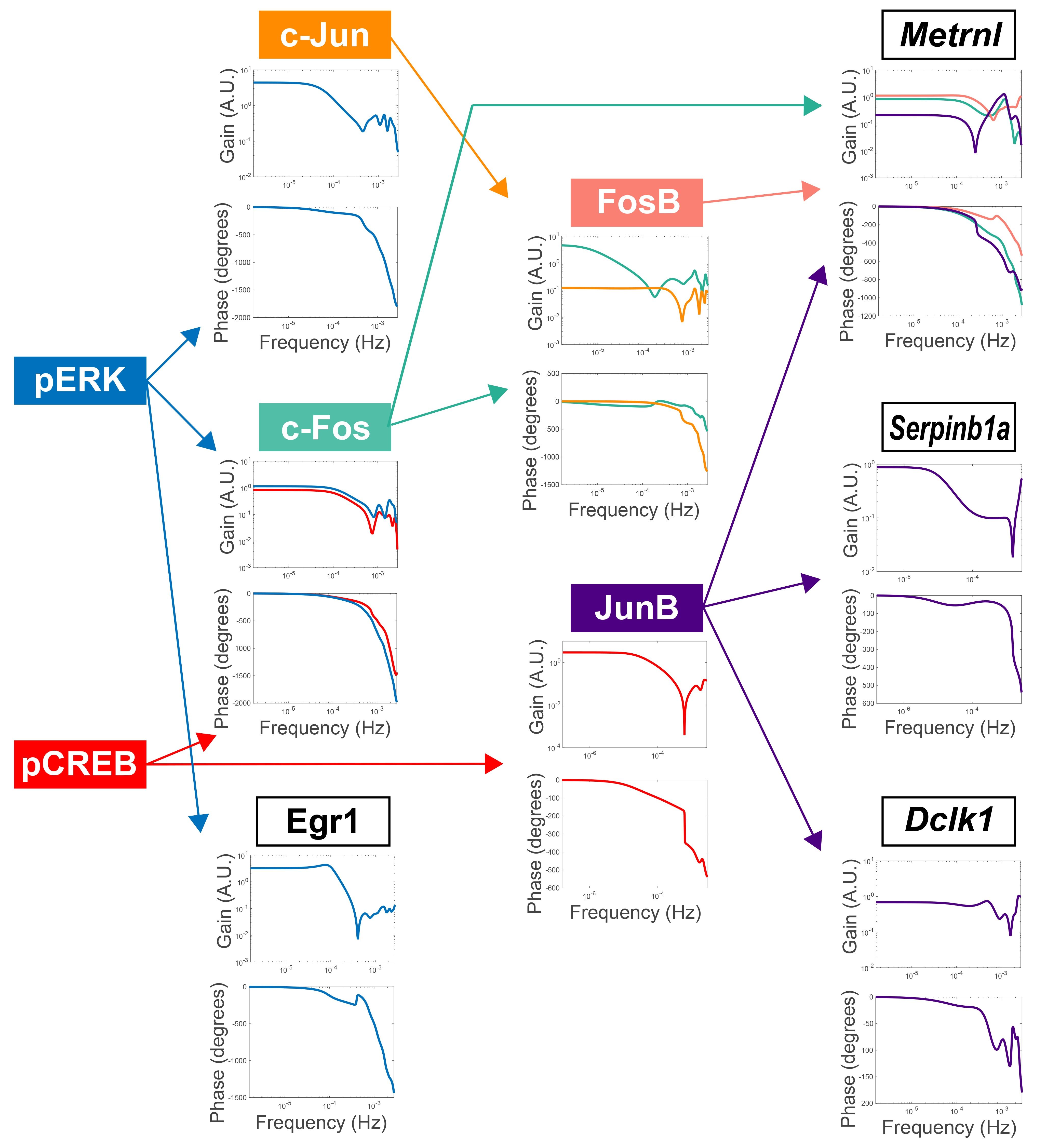

Supplement: S4 Fig — Related to “Calculation of gain and time constant from the linear ARX model” section in Materials and methods. The frequency response curves (upper panel for each) and phase plots (lower panel for each) of the identified linear ARX models in Fig 5A–5C are shown for each output. Arrows indicate the identified I-O relationships in Fig 5A–5C. The colors of the arrows and plotted lines indicate the same input molecules, respectively. Gains and time constants of the linear ARX model are shown in Table 1. Filter characteristics of frequency response curves of these outputs showed low-pass filter characteristics. (TIF) [file pcbi.1005913.s004.tif]
